# Doxycycline Interferes with Zika Virus Serine Protease and Inhibits Virus Replication in Human Skin Fibroblasts

**DOI:** 10.3390/molecules26144321

**Published:** 2021-07-16

**Authors:** Teow Chong Teoh, Sawsam J. Al-Harbi, Ammar Yasir Abdulrahman, Hussin A. Rothan

**Affiliations:** 1 Bioinformatics Programme, Institute of Biological Sciences, Faculty of Science, Kuala Lumpur 50603, Malaysia; ttchong@um.edu.my; 2 Department of Anatomy, College of Medicine, University of Babylon, Babylon, Iraq; drsawsamjasiem@gmail.com; 3 Department of Molecular Medicine, Faculty of Medicine, University of Malaya, Kuala Lumpur 50603, Malaysia; alirhayim83@gmail.com; 4 Department of Biology, College of Arts and Sciences, Georgia State University, Atlanta, GA 30303, USA

**Keywords:** doxycycline, zika virus, flavivirus, NS2B-NS3 viral protease, human skin fibroblast

## Abstract

Zika virus (ZIKV) represents a re-emerging threat to global health due to its association with congenital birth defects. ZIKV NS2B-NS3 protease is crucial for virus replication by cleaving viral polyprotein at various junctions to release viral proteins and cause cytotoxic effects in ZIKV-infected cells. This study characterized the inhibitory effects of doxycycline against ZIKV NS2B-NS3 protease and viral replication in human skin cells. The in silico data showed that doxycycline binds to the active site of ZIKV protease at a low docking energy (−7.8 Kcal/mol) via four hydrogen bonds with the protease residues TYR1130, SER1135, GLY1151, and ASP83. Doxycycline efficiently inhibited viral NS2B-NS3 protease at average human temperature (37 °C) and human temperature with a high fever during virus infection (40 °C). Interestingly, doxycycline showed a higher inhibitory effect at 40 °C (IC50 = 5.3 µM) compared to 37 °C (9.9 µM). The virus replication was considerably reduced by increasing the concentration of doxycycline. An approximately 50% reduction in virus replication was observed at 20 µM of doxycycline. Treatment with 20 µM of doxycycline reduced the cytopathic effects (CPE), and the 40 µM of doxycycline almost eliminated the CPE of human skin cells. This study showed that doxycycline binds to the ZIKV protease and inhibits its catalytic activity at a low micro-molecular concentration range. Treatment of human skin fibroblast with doxycycline eliminated ZIKV infection and protected the cells against the cytopathic effects of the infection.

## 1. Introduction

Zika virus, a flavivirus transmitted by mosquitoes, was discovered in 1947, with no indications that it caused human disease until 1953, when viral infection was confirmed in Nigeria [1]. However, the serosurvey studies showed a wide geographic dissemination of infection with Zika virus, including Egypt, East Africa, Nigeria, India, Thailand, Vietnam, the Philippines, and Malaysia [2,3]. The first considerable outbreak was recorded in 2007 in the States of Micronesia, followed by outbreaks in 2013 and 2014 in French Polynesia and subsequent outbreaks in the Americas and Pacific islands in 2015 and 2016 [1]. The commonly reported symptoms of Zika infection include rash, fever, arthralgia, myalgia, fatigue, headache, and conjunctivitis [2,3,4].

Similar to other flaviviruses, the ZIKV genome consists of a positive-sense single-stranded RNA containing 10,790 nt, encoding 3,419 aa polyprotein. During viral replication, this polyprotein is cleaved by the host cell protease and viral serine protease into the three structural proteins—capsid, membrane, and envelope—that are involved in viral assembly, and seven non-structural proteins—NS1, NS2A, NS2B, NS3, NS4A, NS4B and NS5—that are responsible for viral replication [5,6]. The non-covalent binding between NS2B and NS3 leads to the formation of the NS2B-NS3 protease that cleaves viral polyprotein at junctions between NS2A/NS2B, NS2B/NS3, NS3/NS4A, and NS4B/NS5 proteins and within the capsid, NS2A, and NS4A proteins [7]. Flavivirus proteases are essential for virus replication; therefore, it is considered a target for antiviral drug development [8,9,10].

The incidence of ZIKV outbreaks in the Americas and Pacific islands in 2015 and 2016 has led to the need to find an effective antiviral drug for ZIKV infection. Drug repurposing is a practical approach to finding antiviral drugs to combat emerging virus outbreaks. The main advantages of this approach are a rapid identification, quick clinical trial, and that the selected drug could be available in an adequate quantity for epidemic response. We previously reported that doxycycline interferes with dengue serine protease and inhibits virus replication [11]. As an extension of our investigations on the broad-spectrum antiviral activity of doxycycline, in this study, we evaluated the antiviral activity of doxycycline against Zika virus replication in vitro.

## 2. Results

### 2.1. Doxycycline Binds to the Active Site of ZIKV NS2B-NS3 Protease

**Figure 1:**
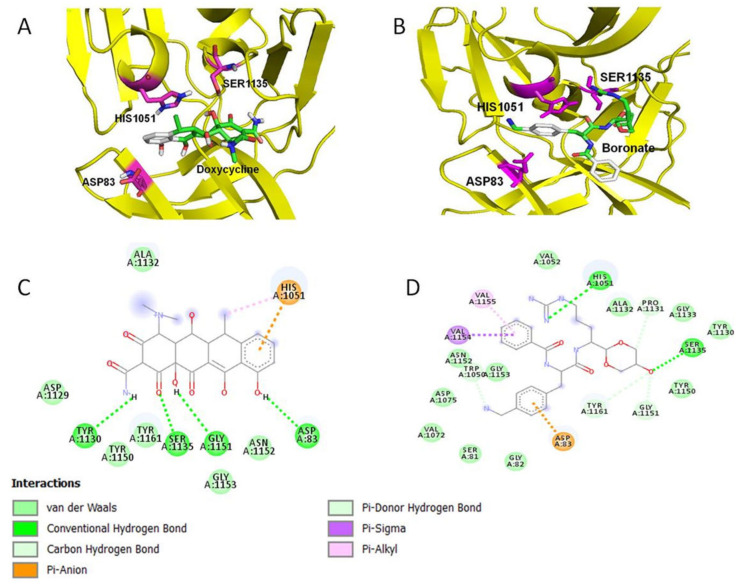
Small-molecule–protein docking of the interaction between doxycycline and ZIKV protease. ZIKA NS2B-NS3pro (PDBID:5lc0) was downloaded from Protein Data Bank (https://www.rcsb.org/, accessed on 5 March 2021) and minimized in Gromacs v5.1.4. using the GROMOS96 43a1 force field, and docking simulations were performed by using AutoDock Vina Version 2.0. (**A**,**B**) Doxycycline and boronate, were docked into the active site of viral protease as a standard. The binding of doxycycline to ZIKA NS2B-NS3 pro is generated by four hydrogen bonds with the protease catalytic triad residues TYR 1130, SER 1135, GLY1151 and ASP83. (**C**) The binding of doxycycline to ZIKA NS2B-NS3 pro is generated by four hydrogen bonds with the protease residues TYR 1130, SER 1135, GLY1151, and ASP83. (**D**) The binding was observed with the boronate but with fewer hydrogen bonds compared to doxycycline.

The interaction between doxycycline and ZIKV protease was identified by an in silico small-molecule–protein-docking study. The ZIKA NS2B-NS3pro (PDBID:5lc0) was downloaded from the Protein Data Bank (https://www.rcsb.org/, accessed on 5 March 2021) and minimized in Gromacs v5.1.4. using the GROMOS96 43a1 force field [12]. The interaction between ZIKV NS2B-NS3pro and doxycycline consists of the putative binding residues of HIS1051, SER1135, and ASP83. Doxycycline and boronate, used as a standard were docked into the active site of viral protease (Figure 1A,B). Both compounds were embedded in the substrate-binding site of ZIKV NS2B-NS3pro. The docking data showed that doxycycline binds with viral protease at a more negative docking energy than boronate (Table 1). The binding of doxycycline to ZIKA NS2B-NS3 pro is generated by four hydrogen bonds with the protease residues TYR 1130, SER 1135, GLY1151, and ASP83 (Figure 1C). Interestingly, doxycycline binds with two of the protease catalytic triad residues, SER1135 and ASP83 by hydrogen bonds. A similar binding was observed with the boronate but with fewer hydrogen bonds and higher docking energy (Figure 1D). Collectively, the in silico data showed that doxycycline exhibited a stronger binding affinity to the ZIKV NS2B-NS3 compared to the standard inhibitor boronate.

### 2.2. Doxycycline Inhibits ZIKV NS2B-NS3 Protease

**Figure 2:**
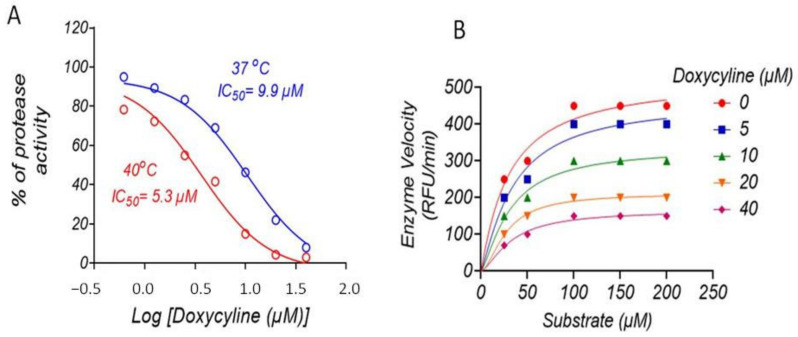
ZIKV NS2B-NS3pro protease assay. ZIKV NS2B-NS3 protease was produced as a recombinant protein by *E. coli*. The kinetic analysis of ZIKV NS2B-NS3 protease was performed in the presence and absence of doxycycline as an inhibitor at four different concentrations. (**A**) Doxycycline efficiently inhibited viral protease at 37 °C and 40 °C, which represent average human temperature (37 °C) and human temperature at a high fever during virus infection (40 °C). (**B**) Kinetic analysis of ZIKV NS2B-NS3 pro was performed in the presence and absence of doxycycline as an inhibitor at four different concentrations.

To validate our in silico data, we sought to test the inhibitory effect of doxycycline on ZIKV NS2B-NS3 protease. First, we produced recombinant NS2B-NS3pro in Escherichia coli ZIKV NS2B-NS3 construct consisting of residues from 49 to 95 of NS2B, linker Gly4-Ser-Gly4 and the N terminus of NS3 (residues from 1 to 170). The enzymatic activity was evaluated by measuring the fluorescence emission from cleaved fluorescence substrate BOC–Gly–Arg–Arg–AMC. Kinetic analysis of ZIKV NS2B-NS3 pro was performed in the presence and absence of doxycycline as an inhibitor at four different concentrations. Doxycycline efficiently inhibited viral protease at 37 °C and 40 °C. These two temperatures represent average human temperature (37 °C) and human temperature at a high fever during virus infection (40 °C). Interestingly, doxycycline showed a higher inhibitory effect at 40 °C (IC50 = 5.3 µM) compared to 37 °C (9.9 µM), as represented in Figure 2A. Furthermore, the results showed that the increasing doxycycline concentrations reduced the maximum reaction velocity (Figure 2B). A considerable reduction in the enzyme velocity was observed at 10 µM of doxycycline that reduced the kinetic activity to 50% (Figure 1B). The concentration at which the enzyme activity was reduced to four times compared to the control was 40 µM. 

### 2.3. Doxycycline Inhibits ZIKV Entry and Replication in Human Dermal Fibroblasts

**Figure 3:**
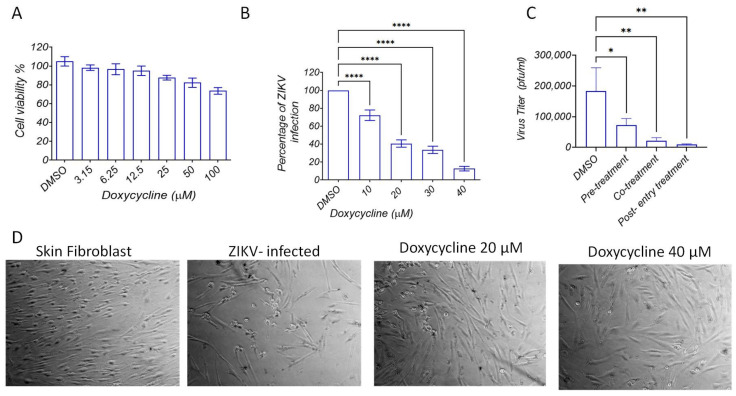
Inhibitory effects of doxycycline against virus replication in human skin fibroblast. (**A**) Cell viability were analyzed by Non-Radioactive Cell Proliferation kit (Promega, USA) according to the manufacturer’s protocols. (**B**) Skin fibroblasts were grown in a 24-well tissue culture plate for 24 h under optimal conditions. The cells were: infected with 0.1 MOI of ZIKV for 2 h, new media containing increasing concentrations of doxycycline were added and incubated for 48 h, and virus quantification was carried out by plaque formation assay using Vero cells. The virus replication was considerably reduced with increasing concentration of doxycycline. Approximately 50% virus reduction was observed at 20 µM of doxycycline. (**C**) Time-of-addition assay showed the potent antiviral activity of doxycycline (40 µM) at the post-entry treatment. (**D**) ZIKV-infection-induced cytopathic effects (CPE) on human skin cells. * *p* < 0.05, ** *p* < 0.001, **** *p* < 0.00001.

To validate our ZIKV protease assay, we sought to test the inhibitory effects of doxycycline against virus replication in human skin fibroblast, one of the ZIKV-permissive cells. The cells were infected with the ZIKV virus at an MOI of 0.1 for 48 h. The virus infection was determined by measuring virus titers in the cell culture supernatant using plaque formation assay. Doxycycline showed no toxicity against human skin fibroblast at the tested concentrations (Figure 3A). The virus replication was considerably reduced with increasing concentrations of doxycycline (Figure 3B). Approximately 50% virus reduction was observed at 20 µM of doxycycline. The time-of-addition assay showed that doxycycline significantly inhibited virus entry, but it was more efficient in inhibiting virus replication (Figure 3C). ZIKV infection induced cytopathic effects (CPE) on human skin cells (Figure 3D). Treatment with 20 µM of doxycycline reduced the CPE, and the 40 µM of doxycycline almost eliminated the CPE of human skin cells after 48 h of treatment. 

## 3. Discussion

ZIKV entry to the skin cells resembles other Flaviviridae family members. The flaviviruses enter skin cells through cellular receptors, enabling migration to the lymph nodes and bloodstream. ZIKV enters human skin fibroblasts, keratinocytes, and immature dendritic cells via adhesion factors such as AXL receptor tyrosine kinase [12,13]. Several factors facilitate ZIKV infection to human skin fibroblasts, including the adhesion receptors and cellular autophagy [12,13]. After cellular entry, flaviviruses typically replicate within endoplasmic reticulum-derived vesicles [14]. Flavivirus replication depends on the enzymatic activities of the viral NS2B-NS3 protease, which represents a central part of the replication complex. ZIKV NS2B-NS3 protease cleaves the viral polyprotein at various junctions to release the structural and non-structural viral proteins [7]. Furthermore, the ZIKV NS2B-NS3 protease can mediate the cytotoxic effects of ZIKV, including delayed cytokinesis and failed mitotic abscission [15]. As such, inhibitors development against the ZIKV NS2B-NS3 protease will be valuable in reducing virus replication and attenuating the cytotoxicity caused by viral protease activity.

This study characterized the inhibitory effects of doxycycline against ZIKV protease and viral replication in human skin cells. We produced catalytically active ZIKV NS2B-NS3 pro as a recombinant protein in *E. coli*. The in silico data showed that doxycycline binds to the catalytic triad of ZIKV protease that consists of Ser1135-His1051-Asp83. Crystallization of this complex has not been successful to date for any flavivirus protease, but it has been shown that a construct comprising ~40 hydrophilic residues of NS2B and ~185 residues of NS3, covalently linked via a Gly4-Ser-Gly4 sequence, displays enzymatic activity [16]. Similar to other flavivirus proteases, such as those of dengue virus (DENV) and West Nile virus (WNV), the ZIKV protease consists of the N-terminal domain of NS3, which carries the catalytic triad Ser135-His51-Asp75, and the co-factor and membrane-bound NS2B that facilitate protease activity and protease integration in the replication complex.

The data of this study also showed that doxycycline inhibited virus protease at low micro-molecular concentrations at normal human temperature and the temperature of human fever. These data indicate that doxycycline binding affinity is stable at fever, and the compounds are efficient in reducing the viral protease activity. Our previous studies also showed that the activity of DENV protease reduced at 40 °C in the presence of protease inhibitors [9]. These data are consistent with the antiviral activity of doxycycline against ZIKV replication in human skin fibroblasts. The compounds showed significant inhibitory effects again virus entry and replication, protecting human skin cells from the cytopathic effects. Previous studies also showed that doxycycline possesses antiviral activities against different viruses such as Dengue virus [11,17], Japanese encephalitis virus [18], chikungunya virus [19], retrovirus [20], and vesicular stomatitis virus [21]. As such, doxycycline has a broad antiviral activity and showed significant inhibitory effects against ZIKV infection.

## 4. Materials and Methods

### 4.1. The Interaction between the Doxycycline and ZIKV NS2B-NS3pro 

The interaction between doxycycline and ZIKV protease was identified by an in silico small-ligand–protein docking study. The ZIKA NS2B-NS3pro (PDBID:5lc0) was downloaded from Protein Data Bank (https://www.rcsb.org/, accessed on 5 March 2021) and minimized in Gromacs v5.1.4. using the GROMOS96 43a1 force field [22]. Docking simulations were performed by using AutoDock Vina Version 2.0 [23,24]. Docked conformations with the most negative docked energy were chosen for further analysis. PyMOL software version 1.3 (PyMOL™ 2010 Schrodinger, LLC) was used to generate the 3D molecular rendering of the docked conformations and the 2-D diagram was computed using Discovery Studio 4.5 Client to analyze the molecular interactions of docked conformations.

### 4.2. Cells and Virus

Human skin fibroblasts were purchased from ATCC (BJ-5ta (ATCC^®^ CRL-4001™) for virus infection. Vero cells (ATCC) were used for virus quantification by plaque assay. The cells were grown in Dulbecco’s modified Eagle’s medium (DMEM) and supplemented with 10% (growth medium) or 2% (maintenance medium) of fetal bovine serum (FBS). The stocks of ZIKV (MR766) were prepared in C6/36 HT cells and titrated by plaque formation in Vero cells.

### 4.3. Production and Purification of ZIKV NS2B-NS3 Protease

The production and purification of ZIKV NS2B-NS3 protease were performed as we described previously [25]. In brief, the C-terminal of the ZIKV NS2B sequence was linked to the *N*-terminal of ZIKV NS3 via an amino acid linker GGGGSGGGG to build up the NS2B-NS3. The DNA construct was synthesized and cloned into a pQE30 expression vector, and the recombinant plasmid was transformed in a DE3 bacterial strain. The recombinant *E. coli* was inoculated in Luria–Bertani liquid medium supplemented with 100 mg/L ampicillin and cultured overnight at 37 °C for recombinant protein production. According to the manufacturer’s instructions, the protein was purified using His GraviTrap ™ Flow precharged Ni Sepharose™ six Fast column (Sigma-Aldrich, St. Louis, MO, USA).

### 4.4. NS2B-NS3pro Protease Assay

Doxycycline was dissolved in DMSO at different concentrations. Recombinant NS2B-NS3pro (0.6 μM) was mixed with the different doxycycline concentrations in 200 mM Tris-HCl pH 8.5. The reaction mixtures were incubated at 37 °C for 10 min, 50 μM fluorogenic BOC–Gly–Arg–Arg–AMC (Peptide Institute Inc., Osaka, Japan) was added into the mixtures, and the reaction mixtures were further incubated at 37 °C for 30 min. Substrate cleavage was detected at Ex360/Em 440 nm in triplicate using a fluorescence spectrophotometer. The IC50 values were determined using non-linear regression models in GraphPad Prism 5.0 software.

### 4.5. Cytotoxicity Assay 

Skin fibroblasts were seeded at 1 × 104 cells/well in 96-well plates and incubated at 37 °C and 5% CO2. The compound was serially diluted in DMEM containing 2% FBS. Each dilution was tested in triplicate. Two controls were included in each experiment culture medium without the compound and culture medium, with different concentrations of the compound in the absence of cells to subtract the background value of the compound in the culture medium. After 48 h, cell viability was analyzed by a Non-Radioactive Cell Proliferation kit (Promega, Wisconsin, USA) according to the manufacturer’s protocols.

### 4.6. Virus Infection and Quantification 

Skin fibroblasts were grown in a 24-well tissue culture plate (1.5 × 10^5^ cells/well) for 24 h under optimal conditions. The cells were infected with 0.1 MOI of ZIKV for 2 h, new media containing increasing concentration of doxycycline were added, and the cells were incubated for 48 h. Virus supernatants were collected and stored in −80 °C. For virus quantification, Vero cells were seeded 0.5 × 10^6^ cells/well in 6-well plates for 24 h. The supernatants-containing viral particles were quickly thawed and diluted 10-fold in serum-free DMEM and 200 μL of the diluted supernatants were added onto the Vero cells’ monolayer for 2 h at 37 °C with gentle shaking every 15 min. The supernatants of uninfected cells were used as the negative control wells. After washing with phosphate-buffered saline, 2 mL of 0.5% agarose overlay diluted in DMEM maintenance medium was added to each well. Viral plaques were stained with crystal violet dye after a 3-day incubation period, as previously described [26,27].

### 4.7. Time-of-Addition Assay

This assay was carried out to test the mode of doxycycline inhibitory effect against ZIKV entry and replication into the target cells. Vero cells were grown in a 24-well tissue culture plate (1.5 × 10^5^ cells/well), infected with ZIKV at 0.1 MOI, and treated with 40 μM of doxycycline. For pre-treatment, the culture media were discarded, and the cells were washed with PBS. Fresh media containing ZIKV and doxycycline were separately added, and the cells were incubated for 1 h at 4 °C. The media were later removed, and the cells were washed extensively with cold PBS to remove unabsorbed virus. Cells were incubated for 48 h and the viral titer was quantified by plaque formation assays. For co-treatment, the mixture of virus supernatant and doxycycline was added onto the cells for 2 h. Then, the mixture was removed, the cells were washed with PBS, fresh media were added, and the cells were incubated for 48 h. The post-entry treatment was carried out by infecting the cells for 2 h, new media containing the compound were added, and the cells were incubated for 48 h.

## 5. Conclusions

This study showed that doxycycline binds to ZIKV protease and inhibits the protease activity at a low range of micro-molecular concentrations. Treatment of human skin fibroblast with doxycycline eliminated ZIKV infection and protected the cells against the cytopathic effects of infection.

## Figures and Tables

**Table 1:** Binding data of doxycycline and boronate to ZIKV NS2B-NS3pro.

Compound	Docked Energy Kcal/mol	H-Bond	vdw	Pi Interactions
Doxycycline	−7.8	4	6	2
Boronate (reference)	−7.2	2	15	3

## Data Availability

All data are presented in this article.
